# Progressive conduction system disease preceding hypertrophy in IVS4+919G>A Fabry variant: a population-based electrocardiographic study

**DOI:** 10.1093/europace/euag258

**Published:** 2026-09-22

**Authors:** Wei-Hsin Chung, Cheng-Chang Tung, Ting-Yuan Liu, Wan-Jung Chang, Kuan-Cheng Chang, Wei-De Lin, Hsin-Yueh Liang, Fuu-Jen Tsai

**Affiliations:** Department of Cardiovascular Medicine, Taichung Municipal Geriatric Rehabilitation General Hospital, Taichung, Taiwan; Division of Cardiology, Department of Medicine, China Medical University Hospital, Taichung, Taiwan; Metal Industries Research and Development Centre, Kaohsiung, Taiwan; Department of Cardiovascular Medicine, Taichung Municipal Geriatric Rehabilitation General Hospital, Taichung, Taiwan; Division of Cardiology, Department of Medicine, China Medical University Hospital, Taichung, Taiwan; Million-person Precision Medicine Initiative, Department of Medical Research, China Medical University Hospital, Taichung, Taiwan; Master Program for Digital Health Innovation, China Medical University, Taichung, Taiwan; Division of Cardiology, Department of Medicine, China Medical University Hospital, Taichung, Taiwan; Department of Cardiovascular Medicine, Taichung Municipal Geriatric Rehabilitation General Hospital, Taichung, Taiwan; Division of Cardiology, Department of Medicine, China Medical University Hospital, Taichung, Taiwan; Department of Medical Research, China Medical University Hospital, Taichung 404332, Taiwan; Genetic Center, Department of Medical Research, China Medical University Hospital, Taichung, Taiwan; School of Post Baccalaureate Chinese Medicine, China Medical University, Taichung, Taiwan; Division of Cardiology, Department of Medicine, China Medical University Hospital, Taichung, Taiwan; Department of Medical Research, China Medical University Hospital, Taichung 404332, Taiwan; School of Chinese Medicine, China Medical University, No. 100, Sec. 1, Jingmao Rd., Beitun Dist., Taichung 406040, Taiwan; Division of Medical Genetics, Children’s Hospital of China Medical University, No. 95, Xueshi Rd., North Dist., Taichung 404332, Taiwan; Department of Medical Laboratory Science and Biotechnology, Asia University, No. 500, Lioufeng Rd., Wufeng Dist., Taichung 413054, Taiwan

**Keywords:** Cardiac Fabry’s disease, Electrocardiography, Right bundle branch block

## Abstract

**Aims:**

The IVS4+919G>A variant is the most prevalent late-onset Fabry variant in East Asian populations, but its electrophysiological trajectory remains incompletely defined.

**Methods and results:**

From a regional genetic screening programme, 905 IVS4+919G>A carriers were identified among 457 581 patients and matched 1:2 to controls by age and sex. Electrocardiographic (ECG) parameters were compared across decades, and echocardiography correlated structural changes with ECG findings. The cumulative prevalence of IVS4+919G>A was 0.198%. Age-specific prevalence in females was ∼2.5-fold higher than in males (0.11–0.15%), without significant change throughout different age categories. Female carriers demonstrated the catch-up phenomenon after the age of 60. Males exhibited a nine-fold risk of right bundle branch block and pacemaker implantation compared with male controls (right bundle branch block, HR 9.286, 95% CI 5.16–16.70, *P* < 0.001; pacemaker implantation, HR 9.197, 95% CI 2.88–29.31, *P* < 0.001), and this precedes the onset of echocardiographic left ventricular hypertrophy. Risk of stroke and atrial fibrillation (HR 1.435, 95% CI 0.69–2.96, *P* = 0.289) was similar between male carriers and controls. PR interval did not shorten but was prolonged instead. Combining left ventricular hypertrophy with conduction abnormalities, ECG detection achieved 88% sensitivity, exceeding echocardiographic criteria alone (51.5%).

**Conclusion:**

The IVS4+919G>A variant is characterized by a progressive electrical phenotype dominated by conduction system disease preceding hypertrophy without excess major arrhythmic events. Electrocardiographic surveillance and phenotype-based risk stratification may be more informative than structural assessment alone for identifying individuals at risk of device therapy.

Institutional Review Board: CMUH113-REC3-076

## Graphical Abstract

**Graphical Abstract:**
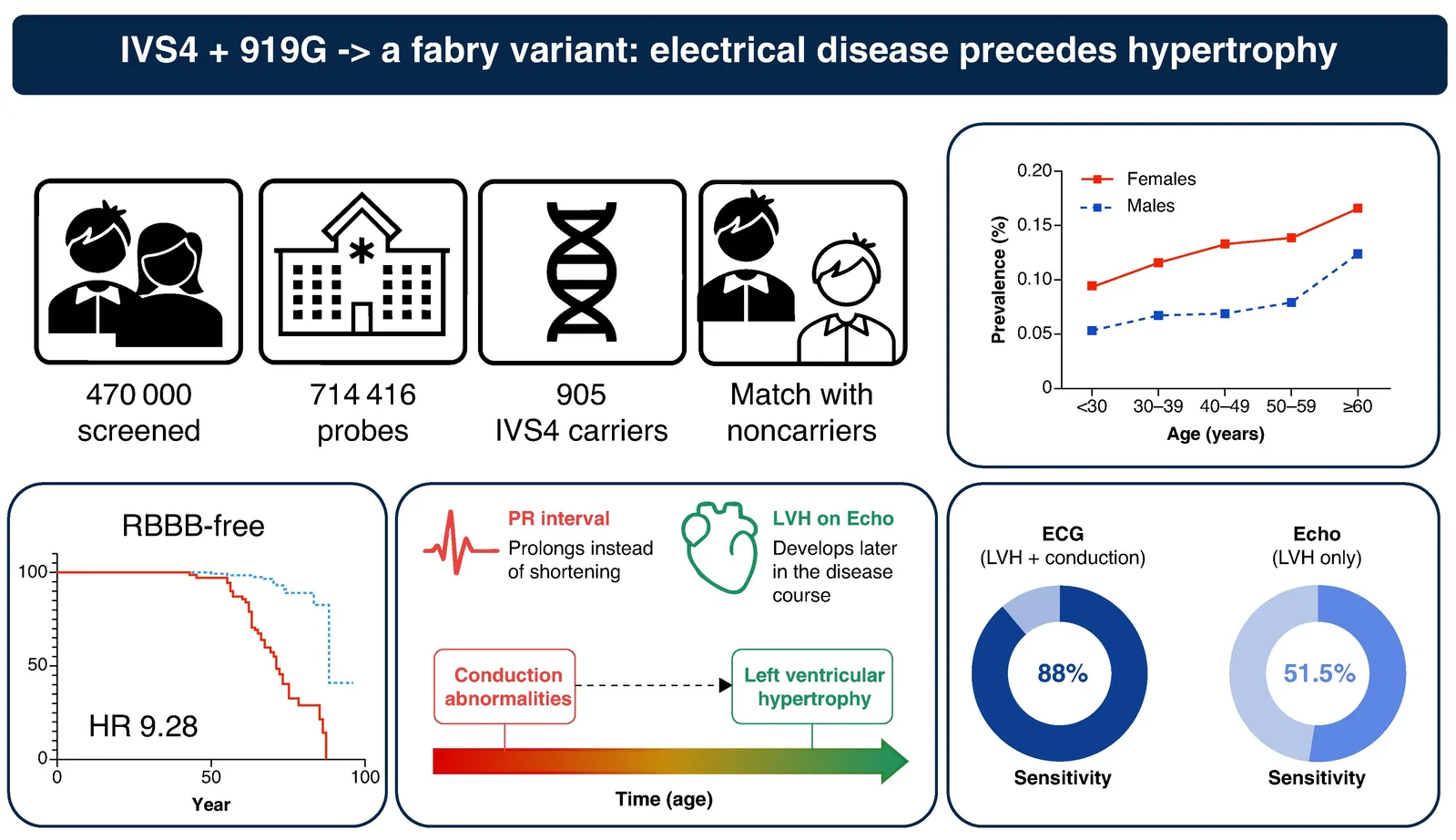

## Introduction

Fabry disease (FD) is an X-linked lysosomal storage disorder caused by α-galactosidase A deficiency, leading to the accumulation of globotriaosylceramide (Gb3) and related glycolipids. The classical form typically presents in childhood with neuropathic pain, angiokeratomas, hypohidrosis, and later renal and cardiac involvement.^1^

In 2002, the deep intronic *GLA* variant IVS4+919G>A (rs199473684; NM_000169.3:c.640-801G>A on the MANE transcript) was linked to cardiac-predominant FD.^2^ This variant introduces a cryptic splice site, leading to pseudoexon inclusion and reduced *α*-galactosidase A activity. With a male prevalence of ∼1 in 875 in Taiwan, it exhibits a strong founder effect among Han Chinese and is frequently encountered in local clinical practice in Taiwan.^3^ Prior small-scale studies characterized this variant as a late-onset phenotype, typically presenting with progressive left ventricular hypertrophy (LVH) and short PR intervals, but with fewer extracardiac symptoms (e.g. angiokeratomas).^4^ However, its natural history, age-stratified progression, and long-term outcomes remain insufficiently defined.^5–7^

Accordingly, this study evaluates the age-stratified prevalence, electrocardiographic (ECG) phenotype, and clinical correlates of IVS4+919G>A in a large genotyped cohort. We specifically aimed to define the temporal progression of conduction disturbances and arrhythmic outcomes to clarify their relevance for electrophysiological risk stratification.

## Methods

### Study design

This study was derived from the Precision Medicine Project at China Medical University Hospital (CMUH), initiated in 2018. CMUH is a tertiary medical centre in Taiwan with ∼1700 inpatient beds and an annual outpatient volume exceeding 3 million visits, serving a geographically diverse population across the country. As previously reported in our recent study,^8^ the cohort includes participants from multiple regions of Taiwan, supporting its broad geographic representation.

All patients presenting to the hospital are invited to participate in the project, and those who consent are consecutively enrolled. Recruitment and sample collection were approved by the Research Ethics Committee of CMUH, Taichung, Taiwan, following the Declaration of Helsinki. Genotyping used a TPMv1 array plate (Thermo Fisher Scientific) with 714 461 probes. Variant calling was performed with Affymetrix Power Tools from 457 581 individuals.^8,9^

For the present analysis, we identified carriers of the intronic *GLA* variant NM_000169.3:c.640-801G>A (IVS4+919G>A, rs199473684) through direct genotyping; these individuals were defined as the IVS4(+) group. Participants were further stratified by age and sex. Clinical data, including electrocardiograms, electronic medical records, and available echocardiographic studies, were systematically reviewed for all IVS4(+) carriers.

A control group was constructed by randomly selecting individuals without the IVS4+919G>A variant at a 2:1 ratio, matched for age and sex.

### Genotyping of *GLA* IVS4+919G>A variant^8^

Peripheral blood was collected during routine evaluations. Genomic DNA was extracted from 200 µL EDTA blood using the MagCore® kit, eluted in 60 µL, and quantified by NanoDrop™ (A260/A280 1.8–2.0). DNA meeting quality criteria was stored at −80°C. Genome-wide genotyping was performed on the Affymetrix Axiom® platform with a custom TPMv1 SNP array (714 461 SNPs selected for Taiwanese allele frequencies). Genotype calls were processed via Affymetrix Genotyping Console and converted to PLINK 1.9 for quality control and downstream analysis.

The *GLA* intronic variant NM_000169.3:c.640-801G>A (IVS4+919 G>A, rs199473684) was directly genotyped. On GRCh38, it is located at X:101399747 C>T (Affx-91439144), creating a cryptic splice site in intron 4 and producing a pseudoexon. Only directly genotyped data were used. Male hemizygotes [IVS4(+) male] and female heterozygotes/homozygotes [IVS4(+) female] were considered IVS4+919G>A-positive.

### Electrocardiographic and echocardiographic acquisition and measurement

Standard 12-lead ECGs (25 mm/s, 10 mm/mV) were analysed via the GE MUSE system by two independent physicians. Left ventricular hypertrophy was defined by Sokolow–Lyon or Cornell voltage criteria. PR interval and QRS duration were measured using standard lead-II and widest-deflection methods, respectively. Right bundle branch block (RBBB) was identified based on QRS ≥120 ms with characteristic morphology (rsr’, rSR’, or notched R in V1/V2; slurred S in I and V6).

Echocardiographic assessments were performed by experienced sonographers using commercially available ultrasound systems (GE Healthcare) in accordance with standardized protocols following the 2015 recommendations by the American Society of Echocardiography and the European Association of Cardiovascular Imaging.^10^ Maximal wall thickness was prioritized for analysis as it is less susceptible to measurement variability than the left ventricular mass index, offering superior sensitivity for early detection of myocardial hypertrophy.

### Statistical analysis

Continuous variables were expressed as mean ± standard deviation or mean ± standard error of the mean, as indicated. Statistical comparison was made using an unpaired Student’s *t*-test (Welch’s *t*-test). Kaplan–Meier event-free and survival analyses were performed using the log-rank test to determine significance. For the time-to-event analysis, only the first event was considered, after which the patients were censored. A *P*-value of <0.05 was considered statistically significant. Data were handled and analysed using Prism version 9 (GraphPad Software, La Jolla, CA).

## Results

Between January 2018 and January 2025, a total of 457 581 patients >20 years old were recruited randomly during their visit to CMUH. Among them, 905 patients were identified with the IVS4+919G>A variant, with a cumulative prevalence of 0.198% (*Figure 1*). Electrocardiogram was retrievable in 481 patients. After age- and sex-matching, another 962 patients were randomly chosen as the control group to investigate the ECG characteristics of the IVS4+919G>A variant.

**Figure 1:**
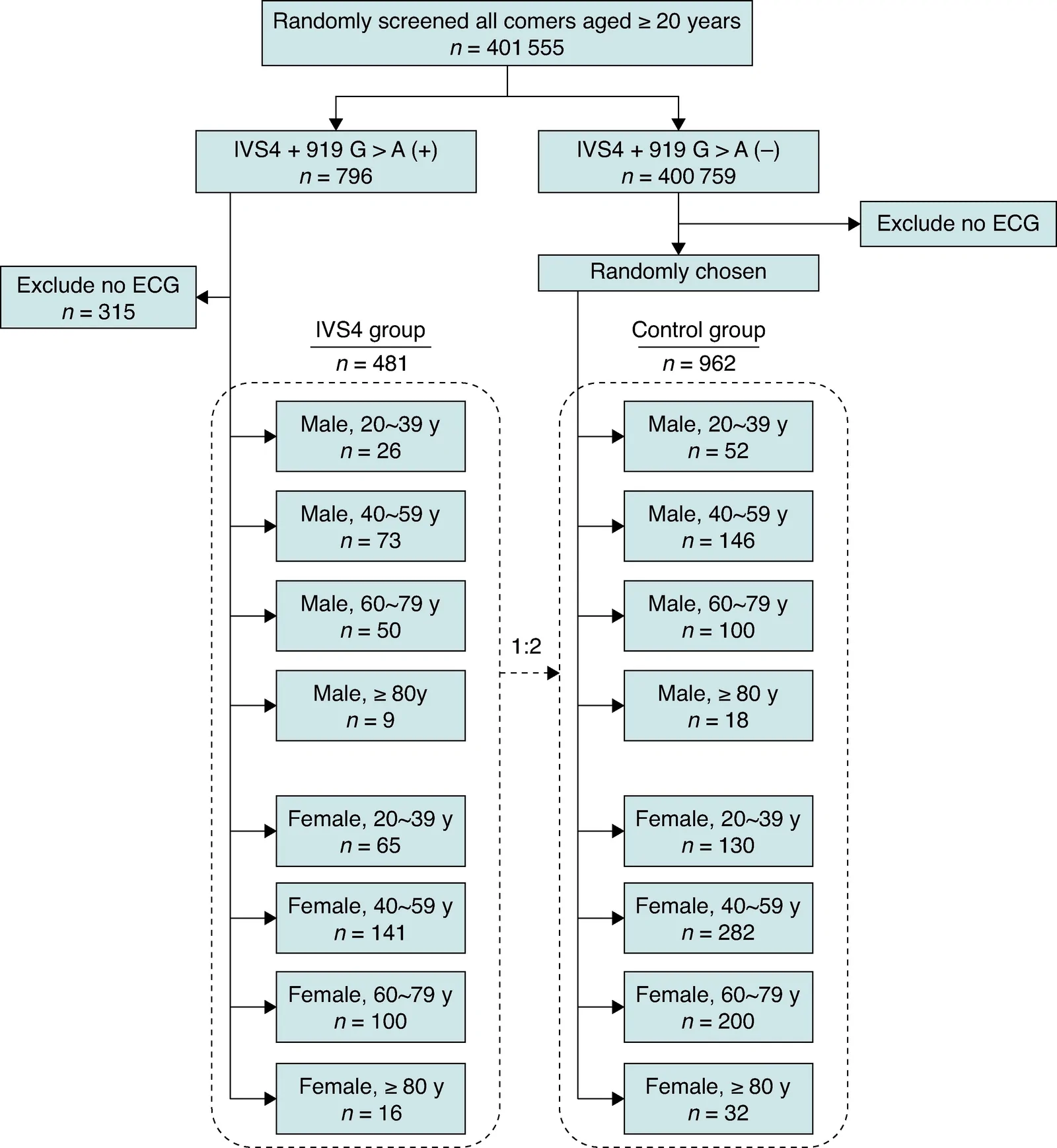
**Diagram of study design.** Death in males: two were attributed to heart failure occurring after the age of 90 years, five were due to malignancy, and a 36-year-old male with a history of alcoholism died from out-of-hospital cardiac arrest. ECG = electrocardiography.

### Stable age-specific prevalence

Among the 457 581 participants, the overall male-to-female ratio was comparable across age groups: 0.98 in those aged 0–19 years, 0.69 in 20–39 years, 0.85 in 40–59 years, 0.99 in 60–79 years, and 0.85 in those ≥80 years (*Table 1*). The age- and sex-specific prevalence of the IVS4+919G>A variant showed a consistent female predominance across all ages. After age 40, the prevalence in both sexes did not change significantly (*Figure 2*). Comorbidities ≥60-year-olds were also comparable between IVS4(+) males and male controls, as in *Table 2* (hypertension 44% vs. 52%, *P* = 0.357; diabetes 22% vs. 30%, *P* = 0.301; coronary artery disease 6% vs. 4%, *P* = 0.585; heart failure 6% vs. 6%, *P* = 1.000; stroke 4% vs. 4%, *P* = 1.000). Notably, end-stage renal disease is significantly more prevalent in controls, suggesting the systemic disease is not elevated in the IVS4(+) group. The similarity was also supported by the comparable medical therapy that the patients were receiving.

**Figure 2:**
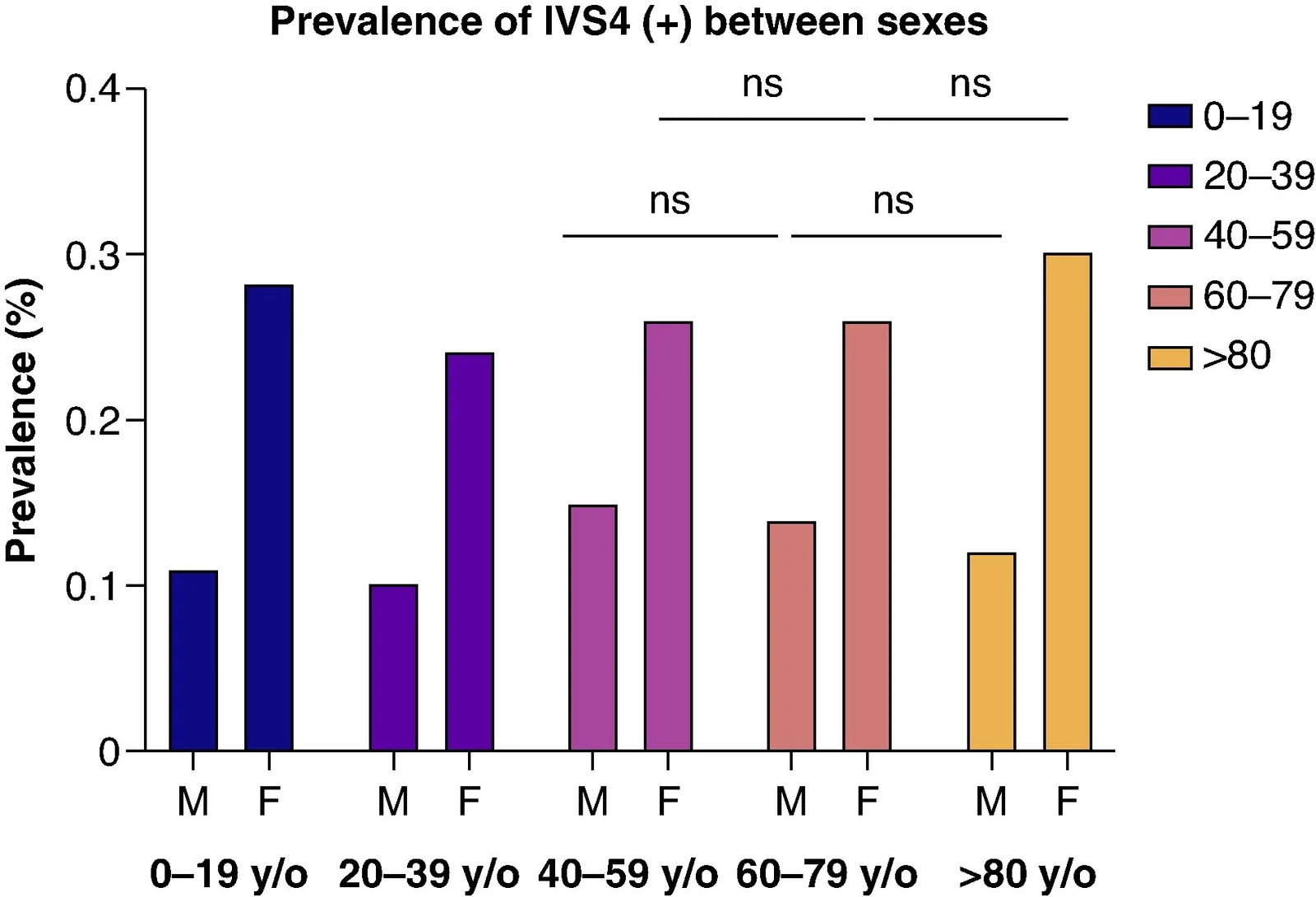
**Prevalence of IVS4+919G>A mutation.** Figure 2 demonstrates the prevalence (%) of the IVS4+919G>A mutation in males and females across different age categories (0–19, 20–39, 40–59, 60–79, and 80–99 years). Female carriers consistently exhibited higher prevalence compared with males in all age groups, with the highest prevalence observed in females aged 80–99 years (0.30%). Note that the prevalence progressively drops in males after the age of 60. F = female; M = male.

**Table 1:** Prevalence of IVS4+919G>A mutation

	Male (*N* = 205 067)					Female (*N* = 247 514)				
Age	0–19	20–39	40–59	60–79	≥80	0–19	20–39	40–59	60–79	≥80
Screened patients	22741	49324	66162	55959	10881	28440	71296	78159	56804	12815
IVS4(+) patients	30 (0.11%)	51 (0.10%)	100 (0.15%)	76 (0.14%)	13 (0.12%)	79 (0.28%)	172 (0.24%)	200 (0.26%)	145 (0.26%)	39 (0.30%)
*P*-value	NA	0.695	0.019	0.647	0.605	NA	0.254	0.439	1.000	0.428

*P*-value compares the prevalence of the IVS4+919G>A variant across adjacent age groups within each sex.

**Table 2:** Demographics of IVS4 and male controls ≥60 years old

60–79 years	IVS4(+) Male (*N* = 50)	Control male (*N* = 100)	*P*-value
HTN	22 (44.0%)	52 (52.0%)	0.357
DM	11(22.0%)	30 (30.0%)	0.301
COPD	3 (6.0%)	4 (4.0%)	0.585
CAD	10 (20.0%)	28 (28.0%)	0.289
HF	3 (6.0%)	6 (6.0%)	1.000
Stroke	2 (4.0%)	4 (4.0%)	1.000
ESRD	0 (0%)	12 (12.0%)	0.011
Medications			
ACEI/ARB	13 (26.0%)	29 (29.0%)	0.706
CCB	13 (26.0%)	27 (27.0%)	0.896
Beta blocker	19 (38.0%)	31 (31.0%)	0.392
Alpha blocker	7 (14.0%)	15 (15.0%)	0.871
Diuretics	6 (12.0%)	6 (6.0%)	0.198
MRA	4 (8.0%)	4 (4.0%)	0.297
ARNI	0	2 (2.0%)	0.315
ADA	5 (10%)	24 (24.0%)	0.043
Antiplatelet	16 (32.0%)	37 (37.0%)	0.545
Statin	14 (28.0%)	35 (35.0%)	0.388
NOAC	2 (4.0%)	9 (9.0%)	0.269
			
≥80 years	IVS4(+) Male (*N* = 9)	Control male (*N* = 18)	*P*-value
Comorbidities			
HTN	5 (55.6%)	14 (77.8%)	0.238
DM	3 (33.3%)	4 (22.2%)	0.553
COPD	3 (33.3%)	2 (11.1%)	0.174
CAD	2 (22.2%)	5 (27.8%)	0.752
HF	3 (33.3%)	3 (16.7%)	0.345
ESRD	0 (0%)	2 (11.1%)	0.308
Medications			
ARB/ACEI	5 (55.6%)	10 (55.6%)	1.000
CCB	3 (33.3%)	13 (72.2%)	0.057
Beta blocker	3 (33.3%)	12 (66.7%)	0.106
Alpha blocker	5 (55.6%)	12 (66.7%)	0.581
Diuretics	5 (55.6%)	5 (27.8%)	0.167
MRA	3 (33.3%)	1 (5.6%)	0.061
ARNI	1 (11.1%)	2 (11.1%)	1.000
ADA	3 (33.3%)	5 (27.8%)	0.772
Antiplatelet	3 (33.3%)	10 (55.6%)	0.283
Statin	3 (33.3%)	8 (44.4%)	0.587
NOAC	5 (55.6%)	8 (44.4%)	0.590

ACEI, angiotensin-converting enzyme inhibitor; ADA, antidiabetic agent; ARB, angiotensin renin blocker; ARNI, angiotensin receptor-neprilysin inhibitor; CAD, coronary artery disease; CCB, calcium channel blocker; COPD, chronic obstructive pulmonary disease; DM, diabetes mellitus; ESRD, end-stage renal disease; HF, heart failure; HTN, hypertension; NOAC, non-vitamin K oral anticoagulant.

### Trajectory of conduction diseases

Among individuals aged 20–39, both the IVS4(+) and control groups showed a low prevalence of abnormal ECGs (see Supplementary material online, *Table S1*, *Figure 3*). During the age of 40–59, IVS4(+) males begin to demonstrate a significantly higher prevalence of RBBB and left anterior fascicular block (LAFB) [IVS4(+) males vs. male controls with RBBB, 12.3% vs. 1.4%, *P* < 0.001; LAFB 5.5% vs. 0%, *P* < 0.001]. The prevalence also progressively increases across other age strata (IVS4 males vs. control males during age 60–79 with RBBB, 34.0% vs. 6.0%, *P* < 0.001; LAFB 20.0% vs. 3.0%, *P* < 0.001; >80 years old with RBBB 44.4% vs. 11.1%, *P* < 0.001; LAFB 44.4% vs. 0%, *P* < 0.001). Notably, left bundle branch block and left posterior fascicular block were not detected in both groups. Permanent pacemaker (PPM) implantation is equally required before the age of 60 [IVS4(+) males vs. control males, 1.4% vs. 0.7%, *P* = 0.612] but became more frequent afterward in IVS4(+) males [IVS4(+) males vs. control males between 60 and 79 years old, 10% vs. 2%, *P* = 0.029; >80 years old, 33.3% vs. 0% *P* = 0.011]. Kaplan–Meier survival analysis demonstrated a nine-time risk of RBBB and PPM implantation in IVS4(+) males compared with controls (RBBB, HR 9.286, 95% CI 5.16–16.70, *P* < 0.001; PPM implantation, HR 9.197, 95% CI 2.88–29.31, *P* < 0.001) (*Figure 4*).

**Figure 3:**
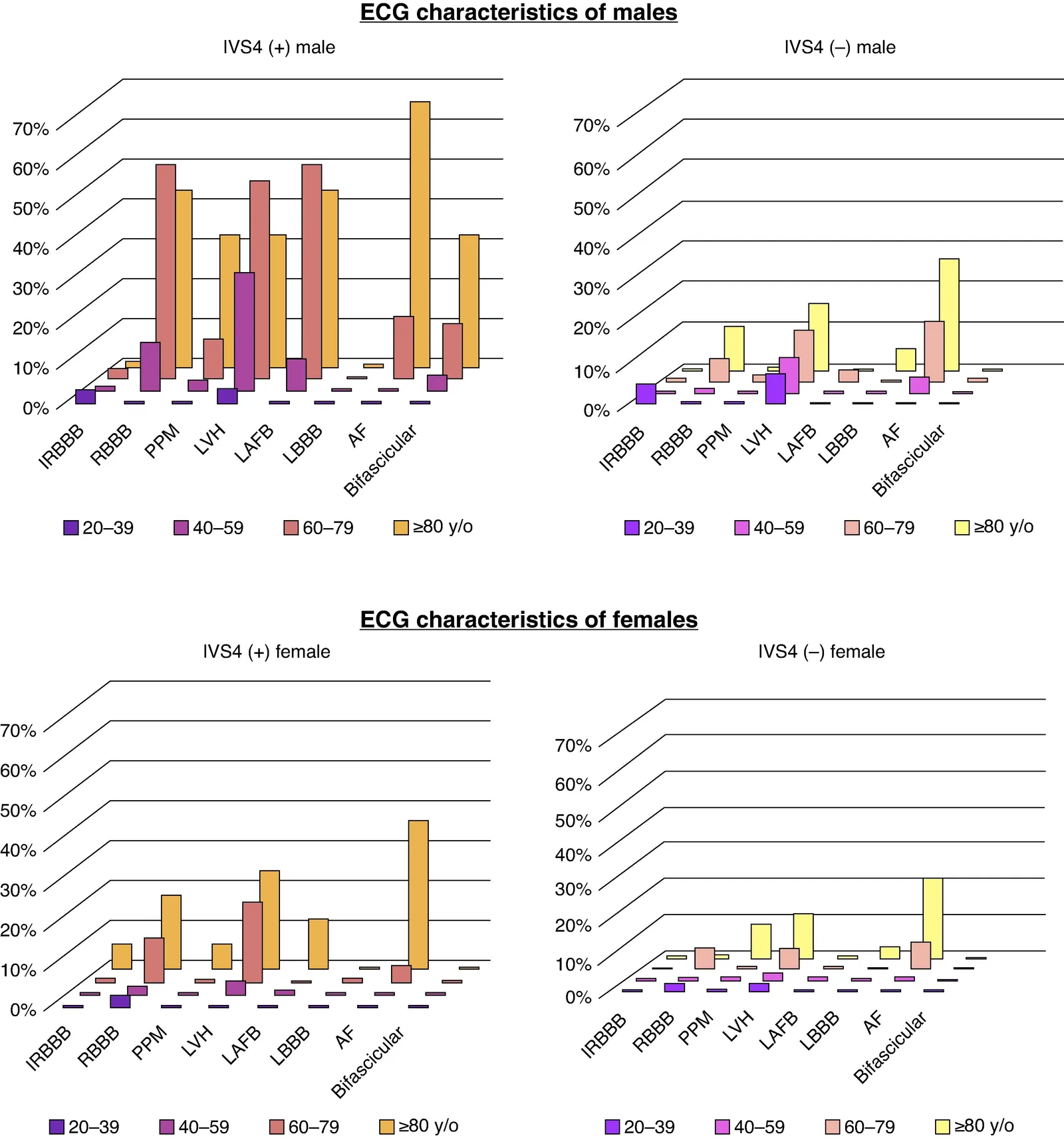
**Electrocardiography characteristics in IVS4+919G>A males and females.**  *Figure 3* illustrates the prevalence of ECG abnormalities between the IVS4+ 919G>A carriers across age groups and sex. Panel *A* demonstrated a higher prevalence of ECG abnormalities in the IVS4+919G>A carriers than in the controls, especially in males. Among IVS4+919G>A carriers, the prevalence of ECG abnormalities, including left ventricular hypertrophy pattern, right bundle branch block, and left anterior fascicular block, increases significantly after the age of 40. Panel *B* provides the comparison showing that ECG abnormalities also increased with age, but to a lesser degree. AF = atrial fibrillation; ECG = electrocardiography; IRBBB = incomplete right bundle branch block; LAFB = left anterior fascicular block; LBBB = left bundle branch block; LVH = left ventricular hypertrophy; PPM = permanent pacemaker; RBBB = right bundle branch block.

**Figure 4:**
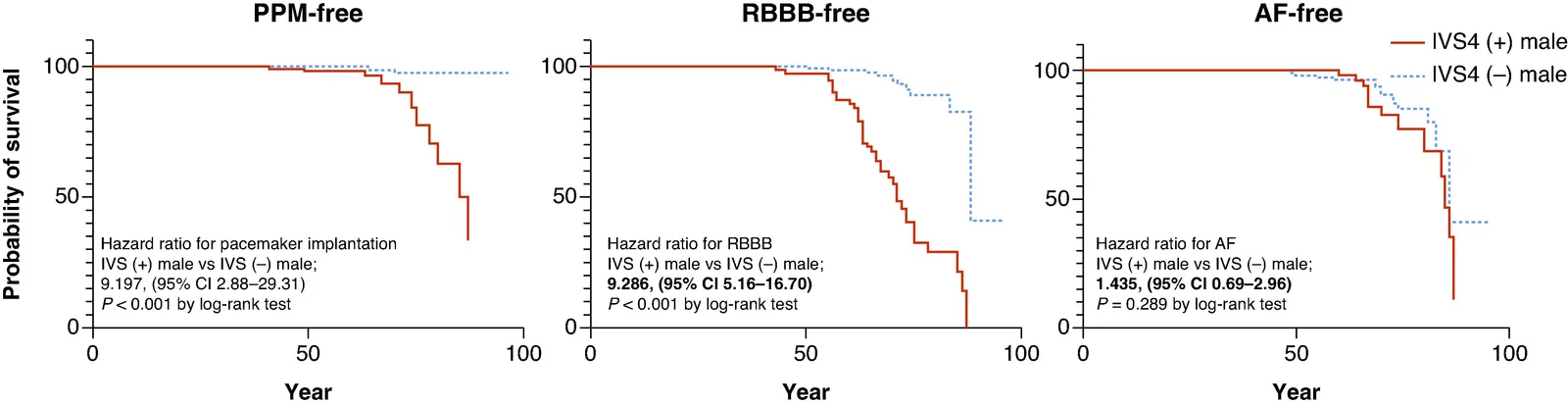
**Kaplan–Meier analysis of conduction diseases and atrial fibrillation.** Figure 4 illustrates the Kaplan–Meier analysis showing that IVS4(+) males demonstrated a significantly higher risk of permanent pacemaker implantation, which was preceded by right bundle branch block. While the hazard ratio for atrial fibrillation was comparable. AF = atrial fibrillation; PPM = permanent pacemaker; RBBB = right bundle branch block.

These findings indicate a progressive, age-dependent conduction phenotype in IVS4+919G>A males, with RBBB and LAFB emerging as the earliest and most penetrant cardiac manifestations.

### Left ventricular hypertrophy and AF as a late phenomenon

Although both groups demonstrated similar prevalence of hypertension, there were significantly more IVS4(+) males who fulfilled the ECG LVH criteria after the age of 40 [IVS4(+) males vs. male control between 40 and 59 years old, 30.1% vs. 8.9%, *P* = 0.001; 60–79 years old, 42.2% vs. 13.9%, *P* = 0.001; >80 years old, 33.3% vs. 16.7%, *P* = 0.337]. In contrast, the prevalence of atrial fibrillation (AF) increases with age in both groups but is comparable before the age of 59 [IVS4(+) males vs. control males between 40 and 59 years old, 0% vs. 2.1%, *P* = 0.213]. After the age of 60, significantly more IVS4(+) males experience AF [IVS4(+) males vs. control males between 60 and 79 years old, 16% vs. 5%, *P* = 0.025; ≥80 years old, 66.7% vs. 22.2%, *P* = 0.026]. However, Kaplan–Meier survival analysis of AF did not differ significantly between groups, as shown in *Figure 4* (HR 1.435, 95% CI 0.69–2.96, *P* = 0.289).

## PR, QRS, and QTc intervals across ages and female ‘catch-up’

PR shortening, a previously reported early ECG feature in classical FD, was not observed in IVS4+919G>A carriers at any age. In IVS4(+) individuals, the PR interval remained comparable across age groups but showed a gradual increasing trend. Among IVS4(+) males vs. females, PR intervals were similar at 20–39 years (149.28 ± 12.9 vs. 144.95 ± 17.4 ms, *P* = 0.391), 60–79 years (157.33 ± 20.0 vs. 153.86 ± 22.5 ms, *P* = 0.357), and ≥80 years (185.00 ± 29.7 vs. 171.80 ± 30.5 ms, *P* = 0.289) but were longer in males at 40–59 years (158.57 ± 26.9 vs. 148.23 ± 19.7 ms, *P* = 0.014) (Supplementary material online, *Figure S1A*). Compared with age-matched controls, IVS4(+) males showed no evidence of PR shortening across all age groups.

In IVS4(+) males, QRS duration was comparable to controls at 20–39 years (92.08 ± 9.6 vs. 93.04 ± 9.4 ms, *P* = 0.391) but became significantly prolonged after age 40 (105.51 ± 19.4 vs. 92.05 ± 10.5 ms, *P* < 0.001). In contrast, QRS duration in females remained similar between IVS4(+) and IVS4(−) individuals across all ages. A similar pattern was observed for QTc in males, which was comparable at 20–39 years (410.54 ± 21.9 vs. 411.44 ± 26.1 ms, *P* = 0.880) but significantly prolonged after age 40 (438.31 ± 27.6 vs. 423.81 ± 22.3 ms, *P* < 0.001) (see Supplementary material online, *Figure S1B*, *S1C*).

### Echocardiographic and electrocardiographic LVH mismatch (*Figure 5*, *Table 3*)

To clarify the correlation between echocardiographic LVH and electrocardiographic LVH, IVS4(+) males aged 60–79 years old were analysed. Thirty-three (66%) patients received echocardiography. Among those, 6/33 (18.1%) showed maximal thickness <10 mm, 10/33 (30.3%) between 10 and 12 mm, and 17/33 (51.5%) with a maximal thickness >12 mm. Importantly, in patients who seemed ‘nonhypertrophic’, 100% demonstrated RBBB. In patients with a maximal thickness of 10–12 mm, 2/10 (20%) did not show any ECG abnormalities. In those with a thickness >12 mm, 7/17 (41.2%) did not fulfil ECG LVH criteria. When combining LVH, RBBB, and LAFB, the sensitivity and specificity of IVS4(+) in males reached 88.0% and 83.0%, while the echocardiographic criteria were 51.5% and 96.3%, respectively (*Table 4*). The result remains unchanged when combining males >80 years old, as in Supplementary material online, *Table S2* and the details in Supplementary material online, *Table S3*. Kaplan–Meier survival analysis demonstrated comparable risk of RBBB and significant LVH (>15 mm), but the development of RBBB precedes LVH by more than 5 years. (*Figure 5*)

**Figure 5:**
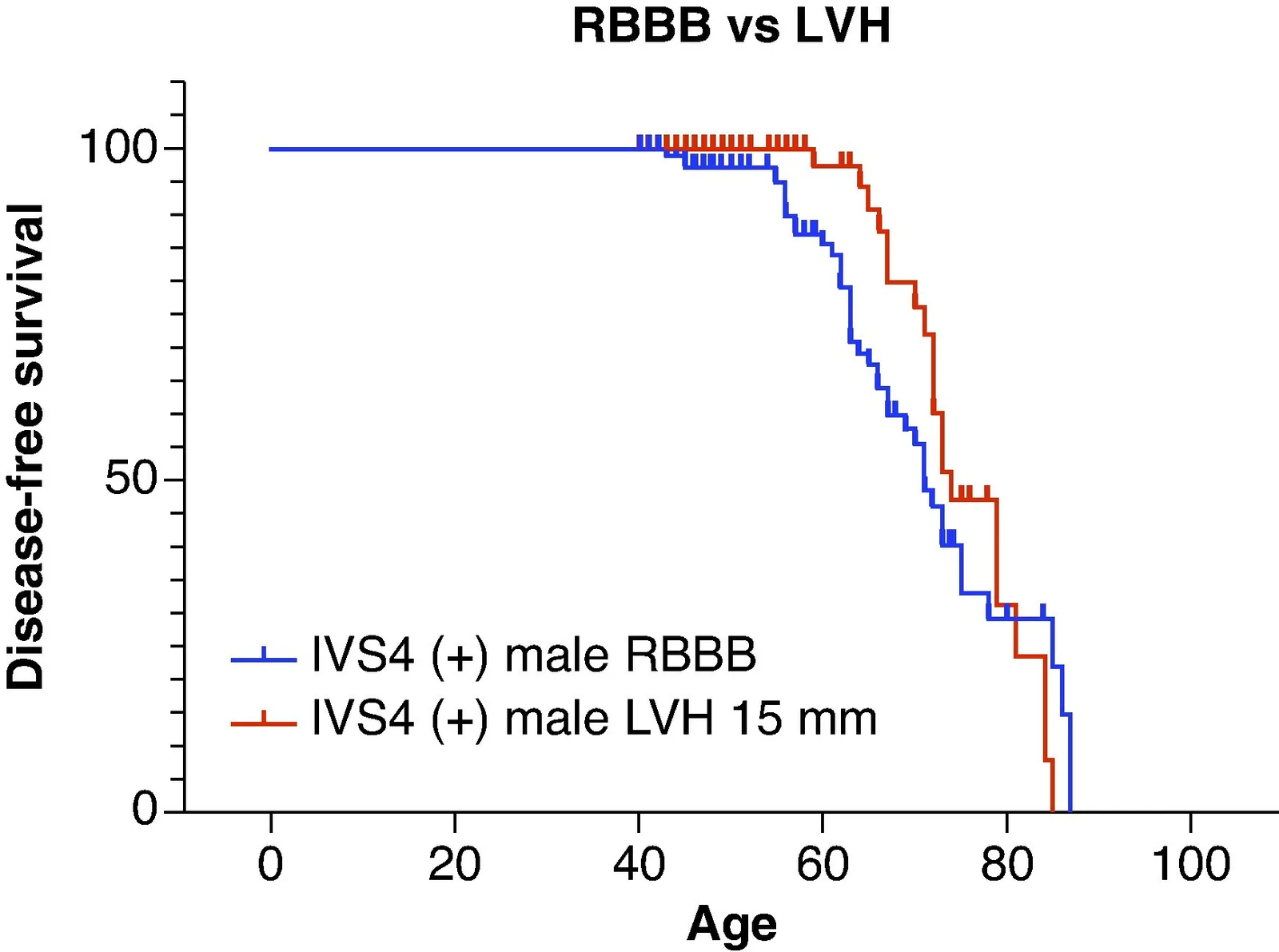
**Kaplan–Meier analysis of conduction diseases and left ventricular hypertrophy.** Figure 5 illustrates the Kaplan–Meier analysis showing the comparable risk of right bundle branch block and left ventricular hypertrophy (>15 mm) in IVS4 (+) males. Note that the onset of right bundle branch block precedes left ventricular hypertrophy by more than 5 years. LVH = left ventricular hypertrophy, RBBB = right bundle branch block.

**Table 3:** Mismatch between echocardiography and electrocardiography

Left ventricle maximal thickness	*N*	Any ECG abnormality^a^ *N* (%)	RBBB *N* (%)	LVH *N* (%)	LAFB *N* (%)
<10 mm	6	6 (100%)	6 (100%)	4 (66.7%)	4 (66.7%)
10–12 mm	6	4 (66.7%)	2 (33.3%)	3 (50%)	4 (66.7%)
>12 mm	21	21 (100%)	13 (61.9%)	13 (61.9%)	14 (66.7%)

Table 3 compares available echocardiography in males >60 years or older. Note that in those with a nonhypertrophic left ventricle, 100% demonstrated RBBB, and in those with significant left ventricular hypertrophy, 40% did not show an LVH pattern on electrocardiography.

^a^If the patients present with RBBB, LVH, or LAFB.

ECG, electrocardiography; LAFB, left anterior fascicular block; LVH, left ventricular hypertrophy; RBBB, right bundle branch block.

**Table 4:** Sensitivity and specificity analysis of echocardiography and electrocardiography

	Sensitivity	Specificity	PPV	NPV
Echocardiographic LVH	51.5%	96.3%	1.95%	99.93%
Electrocardiographic LVH	54%	94%	1.25%	99.93%
RBBB	50.0%	89%	0.63%	99.92%
RBBB or LVH or LAFB	88.0%	88.3%	0.72%	99.98%

LAFB, left anterior fascicular block; LVH, left ventricular hypertrophy; PPV, positive predictive value; NPV, negative predictive value; RBBB, right bundle branch block.

## Discussion

To our knowledge, this is the largest cross-sectional study to investigate the prevalence and ECG characteristics of the IVS4+919G>A variant across all ages, utilizing a sex- and age-matched design. The principal findings include the following: (i) IVS4(+) male carriers develop early-onset conduction abnormalities preceding LVH, and (ii) arrhythmic outcomes and major cardiovascular events do not appear substantially increased relative to controls.

### Attenuated clinical impact of IVS4 variant

In classical FD, cardiac mortality is driven by Gb3 accumulation, triggering inflammation, fibrosis, and eventual heart failure or ventricular arrhythmias. While previous referral-based studies linked the IVS4 variant to high arrhythmogenicity and severe LVH based on different definitions,^11–13^ our population-based cohort reveals a more indolent trajectory. Notably, overt LVH and major adverse cardiac events are not dominant early features. This favourable survival profile is underscored by the complete absence of end-stage renal disease (0% vs. 12% in controls, *P* = 0.011) and no excess cardiac mortality. These findings suggest that the IVS4 variant manifests primarily as a cardiac-tropic conduction disease rather than the systemic, life-threatening progression seen in classical FD.

Furthermore, AF, a key driver of stroke and mortality in typical FD, did not show an increased hazard ratio in our IVS4 cohort.^11,14^ Increased AF prevalence was only observed beyond the eighth decade of life, likely reflecting cumulative age-related remodelling rather than intrinsic arrhythmogenesis. Consequently, stroke and heart failure rates remained comparable to those of age-matched controls. Collectively, our data indicate that while the IVS4 variant leads to progressive conduction disease and increased pacing requirements, it does not carry the high risk of early mortality or systemic organ failure previously reported in smaller cohorts.^15^

### Conduction disease precedes left ventricular hypertrophy as the major presentation

Our study provides compelling evidence that RBBB and LAFB are the primary and progressive manifestations of the IVS4 variant, reflecting a chronic ‘accumulative process’ that begins subtly after age 40. This progressive conduction disturbance eventually leads to a nine-fold increased risk of PPM implantation. Notably, our findings contrast with previously reported electrocardiographic abnormalities, such as PR-interval shortening, left bundle branch block, and left posterior fascicular block, which were rarely observed in this large-scale cohort.^16–19^ Instead, we documented a gradual prolongation of the PR interval and QRS duration. This selective involvement suggests that IVS4-related cardiomyopathy preferentially affects the right bundle branch and left anterior fascicle while sparing the left posterior fascicle. Such a specific pattern may reflect localized intramyocardial fibrosis and region-specific Gb3 deposition, distinguishing it from other classical forms of hypertrophic cardiomyopathy.^20^

Although cardiac magnetic resonance (CMR) imaging is the gold standard for detecting myocardial fibrosis via late gadolinium enhancement, it was not routinely available in our cohort due to regional reimbursement policies and the relatively indolent clinical progression of the IVS4 variant. While this limits detailed substrate-level comparisons, prior literature indicates that a significant proportion of patients with complete RBBB (e.g. 4 out of 11) do not exhibit late gadolinium enhancement, suggesting that conduction abnormalities may precede detectable replacement fibrosis.^21^ This supports our conclusion that LVH alone is insufficient for early disease identification. Consequently, integrating multiple electrocardiographic markers, specifically LVH, LAFB, and RBBB, significantly enhances screening sensitivity, serving as a complementary tool to advanced imaging such as CMR, which provides precise assessment and outcome prediction in FD.^22,23^

### Clinical implications for genotype-guided and phenotype-guided management

While enzyme replacement therapy is established for classical FD after organ involvement,^24–26^ clinical evidence in IVS4 carriers remains limited to structural endpoints. To date, no data demonstrate that enzyme replacement therapy modifies conduction system progression, pacing requirement, or arrhythmic outcomes. Consequently, our findings raise questions regarding the risk-benefit ratio of initiating enzyme replacement therapy in females or patients with milder cardiac phenotypes.^27,28^

There were limited guidelines focusing on managing atypical FD due to a lack of understanding of the natural course of this variant, except for the 2024 Taiwan Society of Cardiology Expert Consensus on Fabry Disease.^29^ Our findings complement the framework in terms of the age- and sex-stratified surveillance strategy. For individuals aged 20–39 years, baseline echocardiography and electrocardiography are recommended, with follow-up every 2–3 years. Between ages 40 and 59, surveillance should increase to every 1–2 years, with consideration of ambulatory ECG monitoring or cardiac magnetic resonance imaging. For those aged ≥60 years, annual cardiac evaluation is warranted.

Surveillance intensity should remain individualized. In patients with LVH, conduction abnormalities, or other evidence of disease progression, more intensive monitoring and timely initiation of disease-specific therapies should be considered. Integration of multimodal assessments, including imaging, electrophysiological evaluation, and biochemical markers such as lyso-Gb3, may further improve risk stratification.

In conclusion, we provide a clinically applicable longitudinal surveillance framework for IVS4 Fabry variant carriers that bridges the gap between consensus recommendations and real-world implementation, particularly in screening-based settings.

### Limitations

Several limitations of this study warrant consideration. First, the cross-sectional design precludes longitudinal observation and systematic tracking of classical extracardiac features (e.g. neuropathic pain, angiokeratoma, and hypohidrosis). Furthermore, because this was a retrospective analysis, genetic segregation data were not systematically available, and healthcare-system barriers to genetic services could not be directly evaluated.^30^ Nonetheless, including multiple age groups provides insights into the natural history of IVS4+919G>A FD using ECG changes as surrogate markers. Second, CMR imaging was not performed, limiting tissue-level characterization. Additionally, 30–40% of ECG and echocardiography data were missing. Because these evaluations were performed at the treating physicians’ discretion, asymptomatic carriers were less likely to be tested. This potential selection bias may overestimate the overall clinical severity. Lastly, the small sample size of the oldest subgroups may limit statistical power, though the markedly pathological changes compared with age-matched controls still capture the core phenotype in advanced age.

## Conclusion

IVS4+919G>A is a late-onset form of FD that predominantly affects males after the age of 60 without increased risk of stroke and AF. RBBB and LAFB precede LVH, which are common but underreported ECG abnormalities. Routine enzyme replacement therapy in IVS4+919G>A carriers without overt organ involvement should be approached with caution.

## Supplementary Material

euag258_Supplementary_Data

## Data Availability

The data underlying this article cannot be shared publicly due to the privacy of individuals who participated in the study. The data will be shared on a reasonable request to the corresponding author.
