# Supplementary material for: Progressive conduction system disease preceding hypertrophy in IVS4+919G>A Fabry variant: a population-based electrocardiographic study
Source: Europace. 2026 Sep 22;28(10):euag258. doi: 10.1093/europace/euag258 (PMC13641066; doi:10.1093/europace/euag258)
Supplement: Supplementary file 1 [file euag258_supplementary_data.docx]

**Progressive Conduction System Disease Preceding Hypertrophy in IVS4+919G>A Fabry Variant: a Population-based Electrocardiographic Study**

Wei-Hsin Chung, MD*^1,2,3^* ; Cheng-Chang Tung, MD*^1,2*^*; *^*^*Ting-Yuan Liu^4,5^ PhD; Wan-Jung Chang MD^2^; Kuan-Cheng Chang MD, PhD*^1,2^*, Wei-De Lin^6-8^ PhD; Hsin-Yueh Liang MD, PhD^2^*^*^*, and Fuu-Jen Tsai^9-12*^ MD, PhD

*^1^Taichung Municipal Geriatric Rehabilitation General Hospital, Taichung, Taiwan.*

*^2^Division of Cardiology, Department of Medicine, China Medical University Hospital, Taichung, Taiwan*

*^3^Metal Industries Research and Development Centre, Kaohsiung, Taiwan*

*^4^Million-person precision medicine initiative, Department of Medical Research, China Medical University Hospital, Taichung, Taiwan*

*^5^Master Program for Digital Health Innovation, China Medical University, Taichung, Taiwan.*

*^6^Department of Medical Research, China Medical University Hospital, Taichung, Taiwan.*

*^7^Genetic Center, Department of Medical Research, China Medical University Hospital, Taiwan.*

*^8^School of Post Baccalaureate Chinese Medicine, China Medical University, Taichung, Taiwan.*

*^9^Department of Medical Research, China Medical University Hospital, Taichung 40402, Taiwan.*

*^10^School of Chinese Medicine, China Medical University, Taichung 406040, Taiwan.*

*^11^Division of Medical Genetics, Children’s Hospital of China Medical University, Taichung 40402, Taiwan.*

*^12^Department of Medical Laboratory Science and Biotechnology, Asia University, Taichung 41300, Taiwan.*

**Supplemental Table 1. Electrocardiography characteristics**

|  |  | **IVS4 (+)** | | | | | | | |
| --- | --- | --- | --- | --- | --- | --- | --- | --- | --- |
|  |  | Male | | | | Female | | | |
|  | Age (N) | 20~39 (N=26) | 40~59 (N=73) | 60~79 (N=50) | ≥80 (N=9) | 20~39 (N=65) | 40~59 (N=141) | 60~79 (N=100) | ≥80 (N=16) |
|  | Sinus | 25 (96.2%) | 70 (100%) | 43 (86.0%) | 2 (22.2%) | 64 (98.5%) | 141 (100%) | 94 (94.0%) | 9 (56.2%) |
|  | AF | 0 (0.0%) | 0 (0.0%) | 8 (16.0%) | 6 (66.7%) | 0 (0.0%) | 0 (0.0%) | 3 (3.0%) | 6 (37.5%) |
|  | iRBBB | 1 (3.8%) | 1 (1.4%) | 1 (2.0%) | 0 (0.0%) | 0 (0.0%) | 0 (0.0%) | 1 (1.0%) | 1 (6.3%) |
|  | RBBB | 0 (0.0%) | 9 (12.3%) | 17 (34.0%) | 4 (44.4%) | 2 (3.1%) | 3 (2.1%) | 11 (11.0%) | 3 (18.8%) |
|  | LBBB | 0 (0.0%) | 0 (0.0%) | 0 (0.0%) | 0 (0.0%) | 0 (0.0%) | 0 (0.0%) | 1 (1.0%) | 0 (0.0%) |
|  | LAFB | 0 (0.0%) | 4 (5.5%) | 10 (20.0%) | 4 (44.4%) | 0 (0.0%) | 2 (1.4%) | 0 (0.0%) | 2 (12.5%) |
|  | LPFB | 0 (0.0%) | 0 (0.0%) | 0 (0.0%) | 0 (0.0%) | 0 (0.0%) | 0 (0.0%) | 0 (0.0%) | 0 (0.0%) |
|  | LVH | 1 (3.8%) | 22 (30.1%) | 21 (42.0%) | 3 (33.3%) | 0 (0.0%) | 5 (3.5%) | 19 (19.0%) | 4 (25%) |
|  | PPM | 0 (0.0%) | 1 (1.4%) | 5 (10.0%) | 3 (33.3%) | 0 (0.0%) | 0 (0.0%) | 0 (0.0%) | 2 (12.5%) |
|  | PR interval (ms) | 149.28±12.9 | 158.57±26.9 | 157.33±20.0 | 185.00±29.7 | 144.95±17.4 | 148.23±19.7 | 153.86±22.5 | 171.80±30.5 |
|  | QTc (ms) | 410.54±21.9 | 438.31±27.6 | 453.47±30.1 | 486.00±49.7 | 426.78±21.8 | 430.74±23.8 | 439.92±28.3 | 447.27±28.0 |
|  | QRSduration (ms) | 92.08±9.6 | 101.51±19.4 | 118.62±22.7 | 136.57±34.8 | 83.59±11.8 | 84.55±10.5 | 89.94±16.4 | 107.20±26.3 |
|  |  |  |  |  |  |  |  |  |  |
|  |  | **IVS4 (-)** | | | | | | | |
|  |  | Male | | | | Female | | | |
| Age (N) | | 20~39 (N=52) | 40~59 (N=146) | 60~79 (N=100) | ≥80 (N=18) | 20~39 (N=130) | 40~59 (N=282) | 60~79 (N=200) | ≥80 (N=32) |
| Sinus | | 52 (100%) | 143 (97.9%) | 93 (93.0%) | 14 (77.8%) | 130 (100%) | 280 (99.3%) | 191 (95.5%) | 24 (75%) |
| AF | | 0 (0.0%) | 3 (2.1%) | 5 (5.0%) | 4 (22.2%) | 0 (0.0%) | 2 (0.7%) | 7 (3.5%) | 5 (15.6%) |
| iRBBB | | 1(1.9%) | 0 (0.0%) | 1 (1.0%) | 0 (0.0%) | 0 (0.0%) | 1 (0.4%) | 0 (0.0%) | 0 (0.0%) |
| RBBB | | 0 (0.0%) | 2 (1.4%) | 6 (6.0%) | 2 (11.1%) | 3 (2.3%) | 3 (1.1%) | 12 (6.0%) | 0 (0.0%) |
| LBBB | | 0 (0.0%) | 0 (0.0%) | 0 (0.0%) | 1 (5.6%) | 0 (0.0%) | 1 (0.4%) | 1 (0.5%) | 1 (3.1%) |
| LAFB | | 0 (0.0%) | 0 (0.0%) | 3 (3.0%) | 0 (0.0%) | 0 (0.0%) | 1 (0.4%) | 2 (1.0%) | 0 (0.0%) |
| LPFB | | 0 (0.0%) | 0 (0.0%) | 0 (0.0%) | 0 (0.0%) | 0 (0.0%) | 3 (1.1%) | 0 (0.0%) | 0 (0.0%) |
| LVH | | 6 (7.7%) | 13 (8.9%) | 13 (13.0%) | 3 (16.7%) | 3 (2.3%) | 6 (2.1%) | 11 (5.5%) | 5 (15.6%) |
| PPM | | 0 (0.0%) | 1 (0.7%) | 2 (2.0%) | 0 (0.0%) | 0 (0.0%) | 0 (0.0%) | 2 (1.0%) | 3 (9.4%) |
| PR interval (ms) | | 153.12±16.5 | 157.92±22.5 | 167.85±32.6 | 172.71±26.0 | 144.72±16.8 | 149.11±19.7 | 159.41±25.3 | 158.50±23.7 |
| QTc (ms) | | 411.44±26.1 | 423.81±22.3 | 433.93±34.7 | 445.61±37.9 | 430.85±25.4 | 432.93±26.0 | 438.88±43.8 | 423.25±74.4 |
| QRS duration (ms) | | 93.04±7.4 | 92.05±10.5 | 93.57±14.0 | 93.11±23.0 | 85.18±11.2 | 83.34±10.1 | 86.50±16.0 | 95.68±16.0 |
|  | |  |  |  |  |  |  |  |  |

AF = atrial fibrillation, LAFB = left anterior fascicular block, LPFB = left posterior fascicular block, LVH = left ventricular hypertrophy, PPM = permanent pacemaker, RBBB = right bundle branch block

**Supplemental Table 2. Mismatch between echocardiography and electrocardiography (including patients >80-year-old)**

| **Left ventricle**  **maximal thickness** | **N** | **Any ECG abnormality***  **N (%)** | **RBBB**  **N (%)** | **LVH**  **N (%)** | **LAFB**  **N (%)** |
| --- | --- | --- | --- | --- | --- |
| <10 mm | 6 | 6 (100%) | 6 (100%) | 4 (66.7%) | 4 (66.7%) |
| 10–12 mm | 6 | 4 (66.7%) | 2 (33.3%) | 3 (50%) | 4 (66.7%) |
| >12 mm | 26 | 26 (100%) | 17 (65.4%) | 16 (61.5%) | 18 (69.2%) |

**Supplemental Table 2** compares available echocardiography in males >60 years or older. Note that in those with a nonhypertrophic left ventricle, 100% demonstrated RBBB, and in those with significant hypertrophy left ventricle, 40% did not show LVH pattern electrocardiography.

* if the patients present RBBB, LVH or LAFB.

ECG = electrocardiography, LAFB = left anterior fascicular block, LVH = left ventricular hypertrophy, RBBB = right bundle branch block

**Supplemental Table 3. Echocardiography and electrocardiography in IVS4+919G>A males > 60 year-olds**

| Patient | Maximal thickness | RBBB | LVH | LAFB |
| --- | --- | --- | --- | --- |
| 1 | 29.8mm | V | V | V |
| 2 | 24.5mm | CAVB | CAVB | CAVB |
| 7 | 11.0mm | V | V | V |
| 8 | 14.3mm |  |  | V |
| 9 | 27.0mm | V |  | V |
| 10 | 11.7mm |  |  |  |
| 11 | 11.8mm | V | V | V |
| 12 | 8.8mm | V | V | V |
| 14 | 18.5mm | V | V |  |
| 15 | 12.8mm |  | V |  |
| 16 | 13.2mm | V |  |  |
| 17 | 9.1mm | V | V | V |
| 19 | 15.2mm |  | V |  |
| 22 | 8.9mm | V |  |  |
| 23 | 14.9mm | V | V | V |
| 24 | 14.9mm |  | V | V |
| 26 | 15mm |  | V | V |
| 27 | 9.2mm | V | V | V |
| 28 | 14.9mm | V | V | V |
| 30 | 12.6mm | V |  | V |
| 31 | 18.9mm | V |  | V |
| 32 | 7.9mm | V |  |  |
| 35 | 24.7mm | V | V |  |
| 36 | 13.6mm |  |  | V |
| 37 | 11.2mm |  |  | V |
| 38 | 9.9mm | V | V | V |
| 40 | 11.3mm |  | V | V |
| 41 | 12.4mm | V |  | V |
| 42 | 12.0mm |  | V |  |
| 43 | 13.2mm | V | V | V |
| 44 | 20.8mm | V | V | V |
| 46 | 12.5mm |  | V | V |
| 47 | 10.9mm |  |  |  |
| 51* | 24.8mm | V |  | V |
| 52* | 17.0mm | V |  |  |
| 56* | 14.5mm |  | V |  |
| 57* | 18.3mm |  | V |  |
| 58* | 17.4mm | V |  | V |
| 59* | 15.6mm | V |  | V |

*Patients number 51-59 were >80 years old.

CAVB = complete atrioventricular block, LAFB = left anterior fascicular block, LVH = left ventricular hypertrophy, RBBB = right bundle branch block.

**Supplemental Figure 1. Electrical alternations**

**Supplemental Figure 1** illustrates the mean PR intervals, QRS and QTc changes across different ages. **Panel A** showed that PR intervals were comparable between groups from ages 20-59 in males, became significantly shorter in IVS4+919G>A carriers during ages 60 to 79, and were longer after age 80. **Panel B and C** show that QRS duration and QTc increase significantly in IVS4(+) males after the age of 40.
